# Supplementary material for: Vaccination and Trust in the National Health System among HIV+ Patients: An Italian Cross-Sectional Survey
Source: Vaccines (Basel). 2023 Aug 2;11(8):1315. doi: 10.3390/vaccines11081315 (PMC10458052; doi:10.3390/vaccines11081315)
Supplement: Supplementary file 1 [file vaccines-11-01315-s001.zip › File S1.pdf]

SERVIZIO SANITARIO NAZIONALE
REGIONE PIEMONTE
Azienda Sanitaria Locale "Città di Torino"
Costituita con D.P.G.R. 13/12/2016 n. 94
Cod. fiscale/P.I 11632570013
Sede legale: Via San Secondo, 29 – 10128 Torino
011/5661566 ☏ 011/4393111

Questionario di rilevazione delle conoscenze in ambito vaccinale nei soggetti HIV-positivi

1) **Cittadinanza**

❑ Italiana ❑ Altro specificare………………………………………

2) **Età** |__|__|

3) **Genere**

- ❑ Uomo
- ❑ Donna
- ❑ Non binario/terzo genere/altro

3) **Stato civile**

- ❑ Libero/a o vedovo/a o divorziato/a
- ❑ Coniugato/a o convivente

4) **Titolo di studio**

- ❑ Nessuno
- ❑ Elementare
- ❑ Medie inferiori
- ❑ Medie superiori/scuole professionali
- ❑ Laurea o superiore

5) **Occupazione attuale**:

- ❑ Casalingo/a
- ❑ Dirigente – Imprenditore/imprenditrice
- ❑ Impiegato/a - Insegnante
- ❑ In cerca di occupazione
- ❑ Libero/a professionista
- ❑ Operaio/a – Artigiano/a
- ❑ Professionista Sanitario
- ❑ Studente/essa
- ❑ Altro (specificare……………………….)

6) **In questo periodo della sua vita è attratto/ha rapporti sessuali**

- ❑ Solo con uomini
- ❑ Prevalentemente con uomini
- ❑ In eguale misura con uomini e donne
- ❑ Prevalentemente con donne
- ❑ Solo con donne
- ❑ Né con uomini né con donne
- ❑ Preferisco non rispondere

7) **Negli ultimi sei mesi ha avuto rapporti sessuali non protetti**

- ❑ Sì
- ❑ No
- ❑ Preferisco non rispondere

8) **Negli ultimi sei mesi ha fatto uso di sostanze stupefacenti iniettive**

- ❑ Sì
- ❑ No
- ❑ Preferisco non rispondere

9) **Ha ricevuto informazioni riguardo i vaccini da personale sanitario?**

- ❑ Sì
- ❑ No

9bis) **Se sì, da chi ha ricevuto informazioni (possibile più di una risposta)?**

- ❑ Ambulatorio HIV
- ❑ Centro Vaccinale
- ❑ Medico di Base
- ❑ Altro (specificare): …………………………..

10) **Si è informato/a autonomamente sui vaccini?**

- ❑ Sì
- ❑ No

10bis) **Se sì, dove ha ricercato informazioni (possibile più di una risposta)?**

- ❑ Internet e social media
- ❑ Televisione
- ❑ Riviste e siti istituzionali
- ❑ Amici e passaparola
- ❑ Altro (specificare): …………………………..

11) **Ha effettuato esami per valutare la possibilità di contrarre qualche malattia prevenibile con vaccino?**

- ❑ Sì
- ❑ No

11bis) **Se sì, quali?**

____
____
____

12) **Per quale delle seguenti delle patologie è stato/a vaccinato/a ?**

		**Sì**	**No**	**Non so/Non ricordo**
12a	*Epatite A*			
12b	*Epatite B*			
12c	*Tetano (almeno un richiamo negli ultimi 10 anni)*			
12d	*Difterite*			
12e	*Pertosse*			
12f	*HPV (Papilloma)*			
12g	*Pneumococco*			
12h	*Influenza (nell'ultima stagione influenzale)*			
12i	*Varicella*			
12l	*Morbillo*			
12m	*Parotite (Orecchioni)*			
12n	*Rosolia*			
12o	*Poliomielite*			
12p	*Meningococco*			
12q	*Haemophilus Influenzae Tipo B (causa di meningite, osteomielite, pericardite…)*			
12r	*Herpes Zoster (Fuoco di Sant'Antonio)*			
12s	*Covid-19*			
12t	*Altro (Specificare)*_____________			

13) **Ha intenzione di vaccinarsi per le seguenti patologie ?**

		**Sì**	**No**	**Non so**	**Sono già vaccinato/a**
13a	*Epatite A*				
13b	*Epatite B*				
13c	*Tetano*				
13d	*Difterite*				
13e	*Pertosse*				
13f	*HPV (Papilloma)*				
13g	*Pneumococco*				
13h	*Influenza*				
13i	*Varicella*				
13l	*Morbillo*				
13m	*Parotite (Orecchioni)*				
13n	*Rosolia*				
13o	*Poliomielite*				
13p	*Meningococco*				
13q	*Haemophilus Influenzae Tipo B (causa di meningite, osteomielite, pericardite…)*				
13r	*Herpes Zoster (Fuoco di Sant'Antonio)*				
13s	*Covid-19*				
13t	*Altro (Specificare)______________*				

14) Quanto concorda con le seguenti affermazioni?

	Per nulla	Poco	Abbastanza	Molto
14a) Credo nelle informazioni fornite dagli operatori sanitari				
14b) Gli operatori del Sistema Sanitario Nazionale sono preparati e aggiornati sulle vaccinazioni				
14c) Ho più fiducia in operatori esterni al Sistema Sanitario Nazionale				
14d) Chi non vaccina viene colpevolizzato dagli operatori del Sistema Sanitario Nazionale				
14e) L'organizzazione sanitaria è flessibile nel modificare tempi e modi delle vaccinazioni su richiesta				
14f) I vaccini servono più al bene della comunità che al bene del singolo				
14g) Gli operatori del Sistema Sanitario Nazionale hanno interesse economico nelle vaccinazioni				
14h) Gli operatori del Sistema Sanitario Nazionale danno informazioni solo sui benefici e non sui rischi dei vaccini				
14i) I vaccini sono un'imposizione e non una libera scelta				

	**A. Ritiene di poter contrarre le seguenti malattie?**				**B. Quanto ritiene gravi o pericolose le seguenti malattie?**			
	**Per nulla**	**Poco**	**Abbastanza**	**Molto**	**Per nulla**	**Poco**	**Abbastanza**	**Molto**
15) Epatite A								
16) Epatite B								
17) Tetano								
18) Difterite								
19) Pertosse								
20) HPV (Papilloma)								
21) Pneumococco								
22) Influenza								
23) Varicella								
24) Morbillo								
25) Parotite (Orecchioni)								
26) Rosolia								
27) Poliomielite								
28) Meningococco								
29) Haemophilus Influenzae Tipo B (causa di meningite, osteomielite, pericardite…)								
30) Herpes Zoster (Fuoco di Sant'Antonio)								
31) Covid-19								

32) **Relativamente alla patologia Covid-19 ritiene che la sieropositività possa metterla a maggior rischio di complicanze?**

- ❑ Sì
- ❑ No
- ❑ Non so/non voglio rispondere

33) **Relativamente alla vaccinazione anti-Covid-19 ritiene che la sieropositività possa metterla a maggior rischio di eventi avversi?**

- ❑ Sì
- ❑ No
- ❑ Non so/non voglio rispondere

Per concludere, le chiederemo alcune informazioni riguardanti la sua condizione. Se non è sicuro delle risposte, può consultare il suo medico.

34) **Da quanti anni è a conoscenza di essere HIV-positivo?**

____

35) **Attualmente assume terapia antiretrovirale?**

- ❑ Sì
- ❑ No
- ❑ Non so/non voglio rispondere

36) **Convive con altre persone sieropositive?**

- ❑ Sì
- ❑ No
- ❑ Non so/non voglio rispondere

37) **Qual è la sua conta linfocitaria?** ________________________________

38) **Qual è la sua carica virale?** ___________________________________

39) **E' affetto da patologie croniche?**

- ❑ Sì
- ❑ No

39bis) **Se sì, quali?**

____

____

Grazie per la collaborazione
